# BET inhibition prevents aberrant RUNX1 and ERG transcription in STAG2 mutant leukaemia cells

**DOI:** 10.1093/jmcb/mjz114

**Published:** 2020-01-03

**Authors:** Jisha Antony, Gregory Gimenez, Terry Taylor, Umaima Khatoon, Robert Day, Ian M Morison, Julia A Horsfield

**Affiliations:** 1 Department of Pathology, Dunedin School of Medicine, University of Otago, Dunedin 9016, New Zealand; 2 Maurice Wilkins Centre for Molecular Biodiscovery, The University of Auckland, Private Bag 92019, Auckland, New Zealand; 3 Southern Community Laboratories, Dunedin 9016, New Zealand; 4 Department of Biochemistry, University of Otago, Dunedin 9054, New Zealand

**Keywords:** cohesin, STAG2, RUNX1, ERG, megakaryocyte, CRISPR-Cas9, chromatin, inducible, enhancer

**Dear Editor,**

Cohesin is a multiprotein complex that not only is essential for cell division but also has key roles in genome organization that underpin its gene regulatory function. Recurrent mutations of genes encoding cohesin subunits occur in myeloid malignancies at 10%–12% (Kon et al., 2013), and the frequency of cohesin mutation in Down syndrome-associated megakaryoblastic leukaemia is even higher (~50%) (Yoshida et al., 2013). Cohesin insufficiency reinforces stem cell programmes and impairs differentiation in haematopoietic stem cells (Mazumdar et al., 2015; Mullenders et al., 2015; Viny et al., 2015). The STAG2 subunit of cohesin is the most frequently mutated in myeloid malignancies (Kon et al., 2013). In contrast to other cohesin subunits, complete loss of STAG2 is tolerated due to partial compensation by STAG1. STAG2 and STAG1 have redundant roles in cell division (Benedetti et al., 2017; van der Lelij et al., 2017). However, cohesin-STAG1 and cohesin-STAG2 have non-redundant roles in facilitating 3D genome organization to delineate tissue-specific gene expression (Kojic et al., 2018).

Cohesin depletion was previously shown to alter chromatin accessibility and transcription of the *RUNX1* and *ERG* genes (Mazumdar et al., 2015), which encode transcription factors that regulate haematopoietic differentiation. Here we used CRISPR-Cas9 to edit K562 erythroleukaemia cells to contain a patient-specific STAG2 R614* mutation (Mullenders et al., 2015) and found that *RUNX1* and *ERG* are precociously transcribed in response to phorbol 12-myristate 13-acetate (PMA)-induced megakaryocytic differentiation.

We characterized two K562 edited lines with homozygous STAG2 R614* mutation (*STAG2-null^A^* and *STAG2-null^B^*) (Figure 1A; Supplementary Figures S1 and S2, Data S1, and Material). Both *STAG2-null* lines showed complete loss of STAG2 (Figure 1B). *STAG2-null* K562 cells exhibited occasional adherent characteristics (Figure 1C) and slower cell cycle progression (Supplementary Figure S3). Array CGH showed that both *STAG2-null* lines had varying minor gains and losses of genetic material relative to the parental line (Supplementary Figure S4 and Data S2). Nevertheless, both *STAG2-null* transcriptomes clustered together and were distinct from the parental line (Supplementary Figure S5). Consistent with potential compensation by STAG1, both *STAG2-null* lines showed 1.6-fold upregulation in *STAG1* (Supplementary Figure S6). Several transcription factors, kinases, chemokines, cytokines, and lineage markers that were lowly expressed in parental cells were significantly upregulated in one or both *STAG2-null* clones (Supplementary Figure S7). Gene set enrichment analyses revealed loss of the typical K562 associated chronic myelogenous transcription profile (Supplementary Figure S8). *STAG2-null* cells upregulated extracellular matrix genes reflecting their adherent phenotype and gained a stem cell-like expression signature (Figure 1D; Supplementary Figure S8). These results show that STAG2 depletion leads to profound morphological and transcriptional changes.

**Figure 1:**
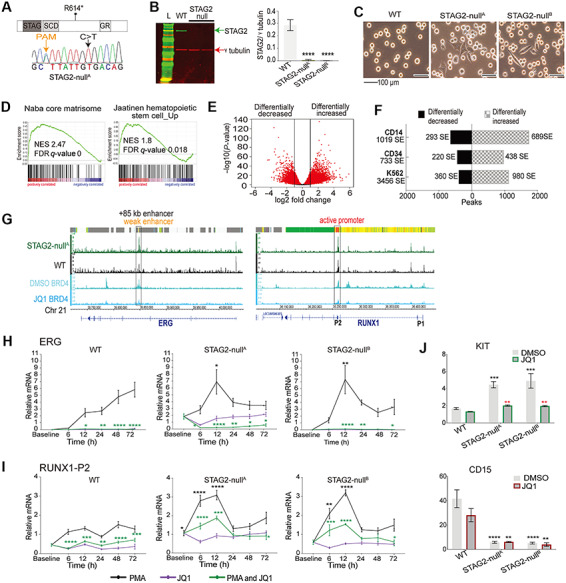
STAG2 mutation alters chromatin accessibility and response to cell signaling. (**A**) Schematic of STAG2 protein showing the position of STAG2 R614* (C>T) mutation. Shown also is the Sanger sequencing plot for CRISPR-Cas9-edited K562 line containing homozygous STAG2 R614* mutation (*STAG2-null^A^*)*.* A silent mutation was introduced at PAM site in *STAG2-null* cells. (**B**) Immunoblot analyses of STAG2 protein levels in parental (WT) and *STAG2-null* cells. Bar graphs show STAG2 protein normalized to γ-tubulin from three biological replicates. Significance was determined by unpaired *t*-test. L, protein ladder. (**C**) Images of WT and *STAG2-null* K562 cells in culture. (**D**) Gene set enrichment analyses showing upregulation of extracellular matrix (Naba core matrisome) and haematopoietic stem cell genes in *STAG2-null^A^.* Shown are the normalized enrichment score (NES) and FDR *q*-value. (**E**) Volcano plot of differential chromatin accessibility in *STAG2-null^A^* compared to WT K562 cells. Significant peaks at adjusted *P*-value ≤ 0.05 are shown in red (52452 sites showing differential accessibility, 29432 differentially increased and 23020 differentially decreased). Lines indicate log2 fold change cut-off: 2. (**F**) Enrichment of differentially increased and decreased accessible sites identified in *STAG2-null^A^* at SEs (defined in K562, CD34^+^ cord blood cells, and CD14^+^ monocytes). (**G**) Integrative genome browser view of normalized ATAC-sequencing signals from *STAG2-null^A^* and WT cells at *ERG* and *RUNX1*. Significant (*P* ≤ 0.05) accessible sites at *RUNX1-P2* promoter and *ERG* +85 kb enhancer are boxed. ChromHMM data for K562 (derived from ENCODE) is shown at the top of each plot, and additional tracks are BRD4 binding in K562 following treatment with dimethyl sulfoxide (DMSO) or 6 h of JQ1 (Liu et al., 2017). (**H** and **I**) *ERG* (**H**) and *RUNX1-P2* (**I**) expression levels examined over a time-course treatment with PMA, JQ1, or a combination of PMA and JQ1. Graphs depict average relative mRNA levels from three biological replicates normalized to two reference genes. Black asterisks denote significant difference between WT and *STAG2-null* lines following PMA-only treatment. Green asterisks denote significant difference between PMA-only and combination of PMA and JQ1 treatment within each cell type. Significance was determined by two-way Anova. (**J**) Relative mean fluorescence intensity (MFI) of KIT and CD15 following treatment with control DMSO or JQ1 for 24 h. Relative MFI for each cell type and condition was determined as a ratio of MFI in stained/unstained. Graphs represent the average of three biological replicates. Significance was determined by two-way Anova. Black asterisks denote significant difference between WT and *STAG2-null* cells for the same condition. Red asterisks denote significant difference between DMSO and JQ1 treatment within each cell type. *P < 0.05, **P < 0.01, ***P < 0.001, ****P < 0.0001.

ATAC-sequencing showed that chromatin accessibility was differentially altered at ~50000 sites in *STAG2-null^A^* cells (Figure 1E; Supplementary Data S3). Motif analyses of differentially accessible sites identified strong enrichment for the enhancer-regulating bZIP or AP-1 factors (FRA1, FRA2, JUN-AP1) at sites of increased accessibility and for CTCF and CTCFL (BORIS) at sites of decreased accessibility (Supplementary Figure S9). In *STAG2-null* cells, we observed increased chromatin accessibility at super enhancers (SEs) defined for K562, CD34^+^ primary cord blood cells, and CD14^+^ monocytes (Figure 1F); 45% genes near SEs with differential accessibility also displayed altered transcript levels in *STAG2-null^A^* cells. SE-proximal genes included those encoding cell lineage markers or transcription factors (Supplementary Figure S10 and Data S4).

The *RUNX1* and *ERG* loci contain SEs in CD34^+^ cells. SEs in proximity to *RUNX1* and *ERG* gained accessibility in *STAG2-null^A^* cells (Supplementary Figure S11). Many of the increased accessible sites were bound by a variety of AP-1 factors at *RUNX1* and primarily by JUND at *ERG* (Supplementary Figure S11). Closer visualization revealed that the prominent ATAC sites in K562 are at the stem cell-associated *ERG* +85 kb enhancer and at the *RUNX1-P2* promoter, and both these sites showed increased accessibility in *STAG2-null^A^* (Figure 1G).

To determine if *STAG2* mutation affects *RUNX1* and *ERG* expression during megakaryocyte differentiation, we stimulated cells with PMA and used quantitative PCR to measure changes over 72 h. Parental K562 cells showed gradual induction of *RUNX1-P1* and *ERG* transcription during stimulation (Supplementary Figure S12; Figure 1H). In contrast, *STAG2*-*null* cells showed an aberrant spike of *RUNX1* transcription 6–12 h post-stimulation from the proximal P2 promoter (Figure 1I; Supplementary Figure S12). A similar aberrant spike was observed in transcription of *ERG* (Figure 1H). By 48 h post-stimulation, *RUNX1* and *ERG* transcription had returned to baseline in *STAG2*-*null* cells. These results imply that increased chromatin accessibility at *RUNX1* and *ERG* in *STAG2-null* cells leads to unrestrained transcription in response to differentiation stimuli. K562 parental cells upregulated *GATA1* and downregulated *KLF1* by 48 h post-stimulation (Supplementary Figure S13), consistent with megakaryocyte differentiation. While *STAG2*-*null* cells successfully downregulated *KLF1*, they were not able to upregulate *GATA1.*

BRD4 is a bromodomain-containing protein that associates with active enhancers (Bhagwat et al., 2016). Notably, BRD4 binds at the *RUNX1-P2* and *ERG* +85 kb enhancer (Figure 1G). JQ1 is a bromodomain and extra-terminal motif (BET) inhibitor protein that reduces BRD4 binding and dampens SE-driven transcription. BRD4 can be removed from *RUNX1* and *ERG* by the BET inhibitor, JQ1 (Figure 1G, data from Liu et al., 2017). We treated *STAG2*-*null* cells with JQ1 together with PMA and measured expression spikes in *RUNX1-2* and *ERG*. JQ1 reduced *RUNX1-P2* and *ERG* expression in parental cells and, strikingly, dampened the PMA-induced transcription spikes in *STAG2-null* cells (Figure 1H and I; Supplementary Figure S12). *RUNX1-P1* transcription was completely blocked by JQ1 in both parental and *STAG2-null* cells (Supplementary Figure S12).

*STAG2-null* cells have reduced expression of the differentiation marker CD15 and elevated levels of the stem cell-associated marker, KIT (CD117), which is only lowly expressed in K562 cells (Figure 1J; Supplementary Figure S14A). Following 24 h of treatment with JQ1, cell surface protein levels of KIT reduced by 2-fold in both *STAG2-null* clones while mRNA was reduced dramatically following 6 h of treatment (Figure 1J; Supplementary Figure S14). However, JQ1 treatment did not increase CD15 in *STAG2-null* cells (Figure 1J; Supplementary Figure S14A), implying that differentiation is not rescued. Collectively, the data indicate that BET inhibition can limit aberrant *RUNX1*/*ERG* transcription and reduce leukaemic stem cell-associated KIT expression in STAG2 mutant cells.

Overall, our results suggest that cohesin-STAG2 depletion de-constrains the chromatin surrounding *RUNX1* and *ERG*, which causes aberrant enhancer-amplified transcription in response to differentiation signals. We show that enhancer suppression using BET inhibitor JQ1 prevents aberrant *RUNX1* and *ERG* signal-induced transcription in STAG2 mutant cells and reduces leukaemic stem cell characteristics of STAG2 mutants.

*[Supplementary material is available at Journal of Molecular Cell Biology online. We would like to thank Catherine Young and Michelle Wilson from the Otago Flow Cytometry Facility (NZ) and Silke Newman from the Department of Pathology, University of Otago (NZ) for assistance and advice on flow cytometry. This work was supported by Health Research Council of NZ award 15/229 to J.A.H. and a Cancer Research Trust of NZ award to J.A*. *and J.A.H. J.A. and J.A.H. designed the research; J.A., T.T., U.K., R.D., and I.M.M. performed experiments; J.A., G.G., and J.A.H. analysed data; and J.A., G.G., and J.A.H. wrote the paper.]*

## Supplementary Material

Antony_et_al_supplementary_material_mjz114

Supplementary_Data1_Differential_genes_STAG2nullversus_WT_mjz114

Supplementary_Data2_Array_CGH_mjz114

Supplementary_Data3_ATAC-seq_differential_STAG2nullA_versus_WT_mjz114

Supplementary_Data4_Superenhancers_mjz114
